# Supporting researcher compliance: assessing institutional readiness for the 2023 NIH Data Management and Sharing Policy

**DOI:** 10.5195/jmla.2026.2361

**Published:** 2026-10-01

**Authors:** Sergio Corrales-Guerrero, Xu Zhang, Thipkanok Wongphothiphan, Jenny Xu, Yungui Huang

**Affiliations:** 1 sergio.corralesguerrero1@nationwidechildrens.org, Project Scientist, Nationwide Children’s Hospital, Columbus, OH; 2 Xu.Zhang@nationwidechildrens.org, Design & Technology Research Manager, Nationwide Children’s Hospital, Columbus, OH; 3 Ping.Wongphothiphan@nationwidechildrens.org, Project Scientist, Nationwide Children’s Hospital, Columbus, OH; 4 Jinyu.Xu@nationwidechildrens.org, Project Scientist, Nationwide Children’s Hospital, Columbus, OH; 5 Yungui.Huang@nationwidechildrens.org, Director, IT Research & Innovation, Nationwide Children’s Hospital, Columbus, OH

**Keywords:** NIH Data Management and Sharing Policy, Research Data Stewardship, Institutional Support, Data Sharing Compliance, Policy Implementation, Researcher Perceptions

## Abstract

**Objective:**

To assess researchers' understanding, readiness, and perceived challenges related to the 2023 NIH Data Management and Sharing (DMS) Policy and identify institutional support needs to facilitate compliance.

**Methods:**

A mixed-method study was conducted using a 15-item survey distributed to 155 researchers at an academic medical center, with 55 responses (35% response rate). The survey evaluated familiarity with the DMS policy, confidence in performing data management and sharing tasks, perceived burden, and desired institutional support. Quantitative data were analyzed descriptively, and open-ended responses underwent inductive thematic analysis to identify key themes.

**Results:**

Most respondents (67%) understood the DMS policy, yet fewer (47%) felt comfortable developing a DMS Plan. Only 29% had used DMPTool.org, while 87% requested tailored templates. Confidence in identifying appropriate repositories (40%) and budgeting for DMS activities (38%) was limited, with 68% perceiving plan execution as burdensome. Qualitative findings revealed two major themes: (1) Operational and Infrastructure Needs; emphasizing the need for centralized consultation, discipline-specific templates, equitable access to expertise, and budget guidance; and (2) Implementation Challenges and Perceived Burden; including perceptions of administrative overload, uncertainty in managing sensitive data, and calls for clearer institutional communication and cultural reframing of data sharing as a value-added activity.

**Conclusions:**

While researchers endorse the principles of the NIH DMS policy, many lack the practical resources and institutional support necessary for effective compliance. Developing centralized infrastructure, standardized guidance, and targeted training may reduce burdens, promote equity, and strengthen a culture of responsible data stewardship. While researchers endorse the principles of the NIH DMS policy, many lack the practical resources and institutional support necessary for effective compliance. Developing centralized infrastructure, standardized guidance, and targeted training may reduce burdens, promote equity, and strengthen a culture of responsible data stewardship.

## INTRODUCTION

In January 2023, the National Institutes of Health (NIH) implemented a policy to enhance the management and sharing of scientific data [1]. This NIH Policy on Data Management and Sharing (DMS) mandates that all NIH-funded research projects include a concise, comprehensive plan for data management and sharing. This policy aims to accelerate the pace of biomedical discoveries, improve research integrity and reproducibility by facilitating the validation of research results, maximize sharing of research by ensuring accessibility to datasets, reduce redundancy in scientific efforts, and boost citizen trust in science [2].

The DMS Policy represents substantive change from the previous 2003 NIH Data Sharing Policy [3], expanding its scope to encompass all NIH-supported research regardless of funding level. By promoting responsible data stewardship, the policy seeks to uphold scientific rigor and integrity while protecting the privacy of human research participants. Researchers are now empowered to choose the most appropriate methods for sharing scientific data, fostering broader collaboration and innovation across the scientific community. Additionally, by ensuring the sharing of scientific data, the NIH anticipates an increase in the reusability of scientific data [4]. Before DMS policy implementation, up to 93% of researchers failed to comply with their own data sharing statements, in a variety of ways such as unresponsiveness, stating that they no longer have access to the data or simply rejecting the data request without providing a reason, among other reasons [5–7]. Consequently, adherence to the policy is expected to prompt changes within the scientific community [8].

A key aspect of the 2023 NIH DMS Policy is its alignment with the FAIR (Findable, Accessible, Interoperable, and Reusable) principles, which provide a framework for enhancing the utility and impact of scientific data [9, 10]. The 2023 NIH DMS policy adheres to these FAIR principles by emphasizing the importance of making data Findable using globally unique identifiers and rich metadata; ensuring that data are Accessible via facilitating seamless and long-term data retrieval; improving Interoperability by using shared vocabularies and formats, enabling data integration across diverse systems and workflows; and underscoring Reusability of data by advocating for detailed documentation and clear usage licenses, thereby maximizing the potential for data to be applied in various research contexts.

Although FAIR data principles have become more widely known since their introduction in 2016, not all research groups have expert data managers or prior experience in sharing their scientific data. Therefore, some teams may lack the necessary knowledge or skills to comply with the FAIR data principles. Acquiring the now-required skills, as well as undertaking the necessary data management and sharing activities to comply with the policy, will require a financial investment from researchers or their institutions. Moreover, the additional preparation of documents, such as DMS Plans, and the need to comply with various and potentially overlapping data-related policies may lead researchers to perceive an increased administrative burden [2]. This explains why the NIH has built an accessible and detailed list with examples of DMS Plans [11], and why online tools such as DMPTool.org-an online platform that contains several plan examples and templates that could be used to build a DMS Plan [12], have been adopted by multiple institutions.

To facilitate DMS policy compliance among researchers at our institute and allocate our institutional resources appropriately, we conducted a mixed-method study to assess researchers’ understanding of the policy, identify factors that may facilitate successful engagement, evaluate their perceived readiness, determine whether the policy is perceived as adding a burden to the research process, and gather their overall opinions on the policy. This will allow an evaluation of potential support needs in training, DMS plan development, data curation, data repository consultation, and other areas. This study shares how we evaluated researchers’ needs and the findings, some of which may benefit other institutions.

## METHODS

To assess the perceived impact of the NIH DMS policy on researchers at our institution and their research activities, we employed a mixed-method approach to collect both quantitative and qualitative data on their understanding of the policy, areas of concern, and suggestions for improvement. Nationwide Children’s Hospital (NCH), with the Abigail Wexner Research Institute (AWRI), is a research-intensive, comprehensive pediatric hospital covering a wide range of pediatric specialties and research fields. This study was determined to not be human research by the Institutional Review Board (IRB) at Nationwide Children’s Hospital (STUDY00004222).

We conducted a survey to evaluate participants’ perspectives on policy requirements, challenges, and support needs. The survey included 14 questions designed to assess researchers’ familiarity with the NIH DMS policy, including DMS Plan requirements and the tools needed to develop such plans (Questions 1 to 4); evaluate researchers’ confidence in performing data management and sharing activities (Questions 5, 6, 8, and 9) and how to do so in compliance with policy requirements (questions 10 to 12); and identify areas where institutional support could help alleviate perceived burdens (Questions 7, 13 and 14). Participants rated their agreement on a 5-point Likert scale, with a “Not Applicable (N/A)” option provided. Responses were categorized as generally positive/agree (ratings of 4 or 5) or generally negative/disagree (ratings of 1 or 2). These questions were developed from previous informal interactions with researchers that had requested assistance to engage with the NIH DMS policy, and analysis of the policy, aiming to keep a short survey to maximize the number of respondents (see Appendix B).

The survey was distributed via email to 155 researchers across the institution, encompassing all members of faculty and staff that were determined to be research leaders at the institution, as they are principal investigators that meet a threshold amount of research funding support. These researchers belong to various fields of medical knowledge, and span basic, pre-clinical/translational and clinical research. Survey results were gathered between May and July 2024.

The survey also included an open-ended question that invited participants to share their perspectives and suggestions for enhancing institutional engagement with the policy, as well as areas requiring additional attention to support understanding and compliance. Open-ended survey responses were analyzed using inductive thematic analysis following Braun and Clare’s six-phase framework [13]. This framework includes familiarization with the data, generation of initial codes, searching for themes, reviewing themes, defining and naming themes, and producing the final analytic narrative. In this process, researchers move from close engagement with the raw data to the development, refinement, and interpretation of themes that capture meaningful patterns across the dataset. The framework was used as an iterative and recursive approach, allowing themes to be reviewed and refined while preserving the depth and context of participants’ perspectives. Primary coding was conducted by a single researcher Thipkanok Wongphothiphan (TW) to support analytic immersion and interpretive consistency across the dataset. To strengthen the credibility and confirmability of the findings, we incorporated a formal audit trail and peer-debriefing/critical audit process with a second researcher Sergio Corrales-Guerrero (SC), who was familiar with the full dataset.

TW first familiarized themselves with the data through repeated readings and memo-writing, documenting preliminary impressions and early pattern candidates. TW then generated inductive, data-driven codes across the full dataset (totaling 13 discrete codes). Coding was flexible and non-exclusive; a single response could be assigned multiple codes where applicable. Codes were then collated into eight candidate themes, which were iteratively reviewed against the coded extracts and the full dataset to assess internal coherence and distinctiveness. Theme names and definitions were refined to capture each theme’s central organizing concept. Finally, the thematic structure was consolidated by organizing the eight themes under two overarching themes comprising eight sub hemes to produce the final thematic map (see Appendix A).

An audit trail was maintained to document analytic decisions and changes across stages (e.g., code generation, code merges/splits, and revisions to theme boundaries and definitions). Following development of the thematic map, SC conducted a critical audit by independently reviewing the proposed codes and theme definitions in relation to the raw survey extracts. TW and SC then held a structured peer-debriefing discussion to examine areas of interpretive divergence and refine theme boundaries and definitions. Revisions arising from this process were documented in the audit trail, and the thematic structure was finalized once themes were clearly defined and adequately supported by the dataset.

## RESULTS

Of the 155 research leaders invited to complete the survey, 55 responded, resulting in a response rate of 35%. In instances where participants submitted multiple entries, only their first submission was retained for analysis. There were five instances of multiple submissions, likely because of the distribution method. An informal comparison of duplicate submissions demonstrated substantial consistency between responses. The first submissions were retained because they more frequently contained comments for Question 15 and generally provided more comprehensive qualitative input.

The survey results revealed that while most researchers reported understanding the requirements of the 2023 NIH DMS policy (67% agreed vs. 13% disagreed), fewer felt confident in developing a DMS Plan, with only 47% expressing comfort compared to 22% who did not. Regarding available resources, the majority of respondents (71%) indicated they had never used DMPTool.org. There was also a strong demand for tailored templates, with 87% of participants expressing interest and only 9% indicating no interest.

When asked about data access, most researchers expressed confidence in their ability to determine which data should be shared (64% agreed vs 6% disagreed). However, confidence was lower regarding control over data access, with only 51% agreeing and 9% disagreeing. This pattern suggests that researchers may find it easier to determine whether data should be shared at all, rather than making more nuanced judgments about conditional sharing and the appropriate levels of access restrictions. Additionally, the majority of respondents (71% agreed vs 12% disagreed) indicated that institutional guidance on data sharing would be beneficial.

Researchers were also asked about their perceived level of expertise in performing tasks required for compliance with the DMS policy. More than half (56%) agreed that they possessed the expertise to document data, metadata, and tools, while 23% disagreed. However, only 45% reported confidence in their ability to curate data, compared to 23% who did not, indicating a gap in perceived preparedness. Furthermore, only 40% of respondents believed they had the expertise to identify an appropriate repository for data sharing, while 27% disagreed. Lastly, when asked whether their team could meet the NIH-required timeline for data sharing, only 39% agreed, and 24% disagreed, suggesting limited confidence in achieving timely data management and sharing compliance.

The final set of questions revealed researchers’ main concerns related to DMS activities. Notably, only 20% expressed they know how to budget for DMS-related costs. While nearly half reported feeling comfortable overseeing their project’s DMS activities (46% agreed vs 22% disagreed), most perceived the execution of a DMS Plan as a significant burden (68% agreed vs 12% disagreed).

As noted above, the survey included an open-ended question inviting researchers to share additional perspectives on the NIH DMS policy and suggestions for how the institution could best support compliance. A subset of 23 respondents provided qualitative feedback. Inductive thematic analysis of these responses revealed two overarching themes [13]: (1) Operational and Infrastructure Needs and (2) Implementation Challenges and Perceived Burden. Each theme comprises several subthemes that collectively illustrate researchers’ perceptions, experiences, and support needs related to the NIH DMS Policy. The percentages in this section are calculated to represent the frequency among open-text answers and not total survey respondents, which can be found in the supplemental materials.

**Figure 1:**
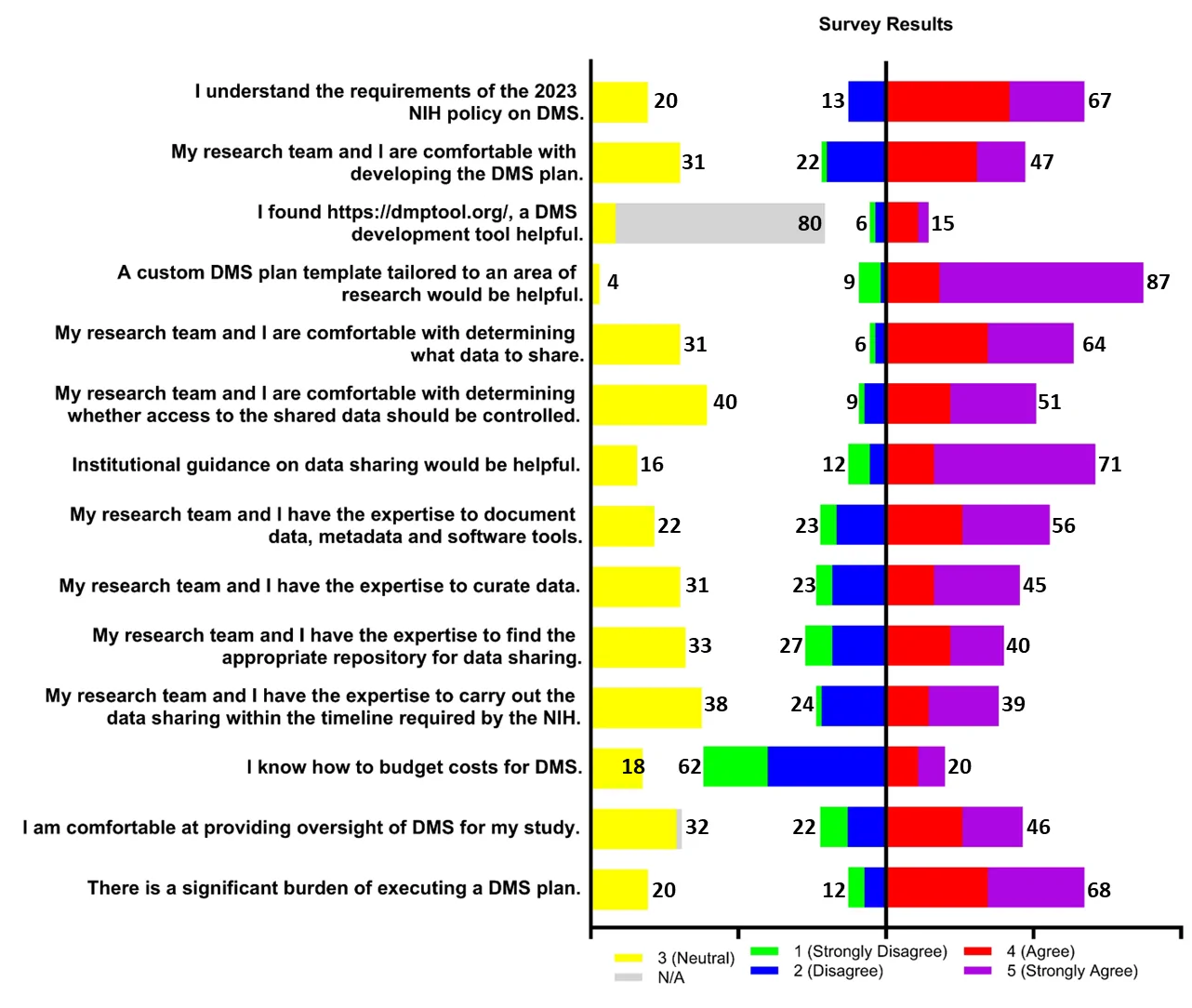
Survey results: Cumulative percentage by response group for each survey question. Represented on the left neutral and N/A responses. On the right a comparison of disagreeing vs agreeing responses. For all questions n = 55, except questions 8 and 13 where n = 54.

**Table 1:** Descending frequency of sub-themes in the open text responses.

Sub-theme	Frequency (%)
1.1 Institutional Infrastructure and Support	65.2
2.2 Data Sharing Complexity	39.1
2.3 Need for Clarity and Communication	39.1
1.2 Desire for Tailored and Practical Resources	26.1
2.1 Burden and Capacity Concerns	26.1
1.4 Financial and Budgetary Constraints	21.7
1.3 Expertise and Equity in Access	13.0
2.4 Cultural and Attitudinal Resistance	8.7

### Theme 1: Operational and Infrastructure Needs

Participants expressed a strong desire for institutional support to facilitate compliance with the NIH DMS policy. This theme encompassed four sub-themes: *Institutional Infrastructure and Support*, *Desire for Tailored and Practical Resources*, *Expertise and Equity in Access*, and *Financial and Budgetary Constraints*.

#### 1.1 Institutional Infrastructure and Support

Researchers want DMS services integrated into institutional infrastructure. This includes staff support, templates, repositories, and consultations.

Approximately 65% of the researchers (n = 15) responding to the open-ended survey question consistently emphasized the need for data management and sharing services to be integrated into institutional infrastructure. Many viewed DMS-related tasks, such as repository selection, metadata documentation, and plan development, as core services that should be supported by trained personnel, rather than managed solely by principal investigators. Participants also highlighted the need for access to consultative services during the grant preparation process. A survey respondent noted:

I wish that AWRI offers: 1) training, 2) templates, 3) personnel supporting writing a DMS plan for my grant applications, 4) identifying an appropriate data repository for my study, and 5) preparing and submitting my data.

#### 1.2 Desire for Tailored and Practical Resources

Participants ask for field-specific templates, curated examples, and applied help with tools like DMPTool.

Over 26% of participants (n = 6) expressed support for the development of customized, discipline-specific resources to assist with both the creation and implementation of DMS plans. Many reported frustration with generic tools and templates, emphasizing the need for curated examples aligned with common study types reviewed by the IRB or representative of the local research context. For example:

The DMPTool is really not that helpful. A simple internal site where someone has curated examples from each of our centers would allow our team to pull parts relevant to their areas and see how others have done this.

#### 1.3 Expertise and Equity in Access

Inequities in access to support are a concern, as is availability of DMS-specific expertise.

Three comments (13%) highlighted disparities in access to DMS-related support across research units. Participants noted that teams within our genomics research center (IGM) benefit from established workflows for depositing data, whereas others face challenges meeting similar requirements without institutional support or repository-specific expertise. These differences raised concerns about equity and consistency in policy compliance. As one survey respondent observed:

Depositing genomic data in NIH repositories is currently very difficult at AWRI for investigators who are not in IGM. Unclear why different rules are applied to investigators in IGM vs. non-IGM investigators.

#### 1.4 Financial and Budgetary Constraints

Concerns about costs, inflexible budgets, and unclear budgeting guidance related to DMS implementation.

Five participants (21.7%) expressed concern about the additional financial burden associated with the DMS policy, particularly within the context of static NIH budget caps. Some described data sharing expenses as unanticipated costs that further strain limited grant budgets. Others highlighted the absence of clear guidance on budgeting for compliant repositories and related services, which complicates financial planning and proposal development. To illustrate:

“Making a dataset ready for sharing is not easy…. It’s a lot of work and therefore not cheap. Unfortunately, we are still living under the $500,000/ year R01 limit that has not increased in decades! Just another expense NIH requires of us but says we must take it out of our allotted budget.”

### Theme 2: Implementation Challenges and Perceived Burden

In addition to structural challenges, participants described the implementation challenges and perceived burdens associated with implementing the DMS policy. This theme includes four sub-themes: *Burden and Capacity Concerns*, *Data Sharing Complexity*, *Need for Clarity and Communication*, and *Cultural and Attitudinal Resistance*.

#### 2.1 Burden and Capacity Concerns

Researchers feel the DMS policy places a high burden on PIs, especially without dedicated support or expertise.

Many researchers (26.1%; n = 6) reported feeling overwhelmed by the new policy requirements, particularly in the absence of dedicated support or prior experience with data sharing. The perceived administrative burden was viewed as detracting from core research activities and contributing to inequities in researchers’ ability to achieve compliance. As one survey respondent noted:

It seems each investigator will invest a significant amount of time developing best practices…I do think some of the burden could be removed from the PI by institutional systems.

#### 2.2 Data Sharing Complexity

Researchers see the technical aspects of preparing and depositing data (especially human data or PHI) as complex and lacking support.

The technical and ethical challenges associated with sharing sensitive or human subjects’ data emerged as a central concern. Almost 40% of participants (n = 9) working with clinical data or protected health information (PHI) expressed uncertainty about how to appropriately de-identify datasets, select compliant repositories, or meet both IRB and NIH requirements. These concerns were particularly salient among researchers studying rare diseases or working with small sample sizes. For example:

The process for human data is very complex and would benefit from a staffed system that would walk investigators through IRB approval for depositing and actual deposit in dbGaP.

#### 2.3 Need for Clarity and Communication

There's a strong desire for clearer institutional guidance and better communication about expectations, tools, and value of DMS.

Several comments (39.1%; n = 9) emphasized the need for clearer institutional communication regarding the expectations, purpose, and procedures associated with the DMS policy. Respondents reported uncertainty about what constitutes compliance, which tools are institutionally supported, and how to navigate overlapping data-related policies.

“Provide templates and better communication. We have sought insight from the OSU medical library because we have not been aware of any resources at NCH.”

#### 2.4 Cultural and Attitudinal Resistance

Some skepticism about institutional motives, DMS value, and overall policy burden reflects resistance to the change.

Finally, two comments (8.7%) reflected underlying skepticism or mistrust toward both the institutional response to the policy and the policy itself. These participants questioned whether data stewardship was being meaningfully prioritized or if compliance efforts were largely symbolic rather than substantive. Others expressed frustration with the top-down implementation of new mandates without corresponding support.

It would be helpful to understand the value of the DMS plan. At the moment, it feels like a tremendous burden that adds nothing to the research enterprise. Understanding that it actually has value, and how to capture that value, would be helpful in motivating adherence to the plan.

## DISCUSSION

This study examined researchers’ perceptions, preparedness, and support needs related to the 2023 NIH DMS Policy within our institutional context. Overall, the findings suggest that while most participants understand the NIH DMS policy, there are several areas of concern regarding how to engage with it. This presents an opportunity for institutional leadership to strengthen and expand existing data management practices, by framing the policy as a means to amplify research impact rather than as an externally imposed mandate, which in turn may further strengthen engagement and compliance.

Despite broad understanding of the policy, many respondents reported gaps in practical knowledge and readiness for compliance. For instance, few expressed confidence in developing or implementing a DMS plan. Specific challenges included data curation, repository selection, budgeting, and meeting NIH-mandated timelines. These findings suggest that understanding the policy is insufficient as there may be barriers for its immediate generalized adoption, and effective compliance requires structured, practical resources that translate requirements into actionable steps. At NCH, addressing these gaps through structured institutional training, technical assistance, and standardized processes could substantially reduce barriers to implementation. Other institutions may likewise benefit from integrating similar resources into their research infrastructures.

A prominent theme emerging from the survey was researchers’ call for a stronger institutional infrastructure to support DMS compliance. Respondents to the open text question stated the need for centralized consultation services during grant preparation, particularly for crafting DMS plans that align with both NIH expectations and local research practices. Such services were viewed as essential for reducing the administrative burden placed on individual investigators. This aligns with the survey results, in which a majority of respondents agreed that institutional guidance would be beneficial, underscoring a clear need for a coordinated institutional response to the challenges associated with policy compliance. Equally important was the call for dedicated personnel with expertise in data management who could provide direct assistance in DMS Plan development and repository selection. In the open text questions 26.1% of open-response participants also underscored the value of institutional templates and curated examples tailored to specific research disciplines and contexts. These comments are consistent with the survey findings, in which the desire for templates was rated very highly, and lack of confidence in selecting repositories showed the second highest level of disagreement, surpassed only by budgeting. Collectively, these requests highlight the importance of embedding DMS expertise within institutional systems rather than relying solely on individual investigators to interpret and operationalize policy requirements.

Another concern identified was the uneven level of preparedness for DMS-related activities across research centers. Three respondents pointed out that certain units, such as those engaged in genomics research, benefit from well-established workflows and familiarity with data repositories, whereas investigators in other departments often lack comparable resources, experience or guidance. This perceived disparity may contribute to perceptions of inequity and inconsistency in institutional DMS policy implementation. This may also challenge notions of fairness and risk undermining collective compliance efforts, as smaller teams or less experienced investigators may face disproportionate administrative and technical burdens. These concerns further highlight the need for support in repository selection, as well as the gaps in expertise related to data curation and the handling and documentation of data, metadata, and associated tools identified in the survey. Addressing this gap will require development of standardized policies and support mechanisms to ensure equitable access to the expertise, tools, and infrastructure necessary for effective data and metadata management, curation, and consequently DMS compliance across all research teams.

A key finding was that researchers perceived the execution of DMS Plans as a substantial burden (68% agreement in survey and 26.1% in open responses), particularly regarding time and financial investment. At NCH, this perception may stem from the combination of budget limits and the limited availability of dedicated data management personnel within many research teams. This aligns with prior literature documenting tension between expanding compliance requirements and constrained grant budgets [2, 8]. Moreover, most survey respondents reported that they do not know how to budget for DMS-related costs, and only one-fifth indicated that they feel comfortable doing so. This further underscores the extent to which budgetary considerations represent a concern for researchers. Consequently, without explicit budgeting strategies and institutional overhead mechanisms to offset associate costs, the policy risks exacerbating inequities by disproportionately straining smaller or less-resourced teams. To mitigate this, NCH and similar institutions should provide clear budgeting guidance for recognizing DMS-related costs in grant applications and develop institutional initiatives that promote data reusability and shared infrastructure.

Participants also identified the complexity of managing sensitive data and the added burden of DMS compliance oversight as major challenges. Investigators working with human subjects or rare disease datasets expressed uncertainty regarding appropriate repository selection and in one comment about data de-identification. These findings may also help explain the relatively low survey scores related to researchers’ confidence in determining whether data access should be restricted or in selecting an appropriate repository. These findings could potentially hint towards agreement with prior publications highlighting heightened concerns about privacy safeguards and intellectual property protections in research contexts [14, 15]. However, as noted earlier, intellectual property and data ownership was not identified among the open-text responses and perhaps these findings are reflective of the overall lack of expertise in repository selection, data curation and potentially participant privacy safekeeping. Within the NCH setting, this underscores the need for additional institutional consultation services, particularly those providing guidance on repository selection, to strengthen researcher confidence and compliance. More broadly, these results suggest that generic federal guidance is insufficient, and institutional-specific support structures are essential to ensure privacy protections, regulatory compliance and researcher trust.

39.1% of participants of the open text response also indicated the need for improved institutional communication and clarity surrounding the DMS policy. Respondents expressed uncertainty about what constitutes compliance, and which institutional tools are officially supported. Such ambiguity may contribute to confusion and potentially resistance to engagement. Researchers suggested that clearer, coordinated communication strategies could reduce these barriers. Moreover, this enables a shift in how the policy is presented: rather than framing it as an external mandate, institutions should position data sharing as an opportunity to enhance research visibility, collaboration, and societal impact. This reframing could reduce potential attitudinal resistance by aligning compliance efforts with researchers’ intrinsic motivations for scientific contribution and innovation, thereby transforming perceived obligations into shared institutional values.

To prepare an institutional response to the publication of the policy, NCH coordinated pre-existing resources, team members and added a new member to lead the response and provide the applicable expertise to help researchers engage with the policy. This led to the development of the present study. In response to the findings above, the team has implemented a suite of new services aimed at facilitating the transition to compliance with the NIH DMS policy. First, a website was developed using the institution’s internal communications platform. The site provides a concise summary of the policy requirements, along with a curated collection of freely available resources, including templates, DMS Plan examples, and checklists.

Survey responses indicated that many researchers had not previously engaged with DMPTool.org, which offers additional tools and guidance for developing Data Management and Sharing (DMS) Plans). In response, the website highlights DMPTool.org and includes a step-by-step guide to support its use. Additionally, our team created and integrated supplementary guidance materials directly within DMPTool.org, ensuring that tailored support is available to researchers as they develop their DMS Plans using the platform.

Additionally, a presentation introducing the policy was developed and delivered through several institutional seminar series. The content was designed to address key concerns raised by researchers, including budgeting for data management and sharing, selecting an appropriate repository, and determining when data should be stored under controlled access. The presentation was recorded, and both the video and slide deck were made available to researchers on the website for ongoing reference. Based on survey responses, we identified the research units with the highest representation and subsequently delivered tailored versions of the presentation to those groups, customizing the content to address their anticipated priorities and most pressing needs.

A consultation service was also established to support researchers seeking assistance with their DMS activities, particularly in the development of DMS Plans. Support is available via email, chat, and video conferencing. As part of the consultation process, a team member may conduct a “discovery” conversation with the researcher to assess their specific needs, identify potential solutions, and clarify relevant policy requirements. These consultations often include assistance with selecting an appropriate repository, connecting researchers to relevant resources, ensuring that proposed data-sharing approaches align with privacy and confidentiality protections, and reviewing or drafting compliant DMS Plans.

To support continuous improvement, a satisfaction survey is distributed following each consultation. In addition to assessing overall satisfaction, the survey gathers feedback on which components of the DMS Plan researchers find most challenging. This allows us to update the recurrent presentations on the policy by further explaining topics where confusion may prevail. Furthermore, several months after NIH proposal submission, researchers are invited to share any feedback received from NIH regarding their DMS Plans. This information enables the consultation service to adapt and remain responsive to evolving NIH priorities and evaluation criteria.

Finally, our institution continues to pursue broader initiatives to address concerns that extend beyond the scope of the services described above, including inequities in access to data management expertise and the inherent complexity of data sharing. These efforts include the development of data literacy training modules and the promotion of initiatives aimed at enhancing data reusability. Collectively, these initiatives represent a proactive institutional strategy to transform researcher concerns into opportunities for strengthening sustainable data stewardship capacity and promoting responsible research data sharing.

This study has several limitations. Although a 35% response rate was achieved, respondents represented a wide range of research units. As a result, subgroup analyses comparing individual units to the overall dataset, or to one another, were not feasible due to limited sample sizes within each unit. Moreover, at the time of this study we were not able to determine whether the number of respondents per research unit was a fair representation of all researchers in the unit, which limits the interpretation of representation by field of research. Additionally, to encourage participation, the survey was intentionally kept brief to minimize the time burden on researchers. Consequently, the scope of questions was limited, and some areas of inquiry that might have provided deeper insight into researchers’ needs and better informed the prioritization of support services were not explored.

Most recently, in May 2026, the NIH introduced a new DMS Plan format. This streamlined and user-friendly format is intended to facilitate the completion of DMS Plans by researchers. Although the revised format may address some of the concerns identified in this study, such as the need for standardized templates, it is important to note that templates were already available at the time the survey was administered. Furthermore, many of the concerns identified in this study extend beyond the creation of the DMS Plan itself and relate to the operational requirements of effectively implementing the policy throughout the research lifecycle and period of performance. These challenges include determining which data should be shared, securing institutional support, determining appropriate data access controls, curating data, developing appropriate budgets, addressing administrative burden, and selecting suitable repositories.

In summary, this study highlights both the opportunities and challenges associated with implementing the NIH DMS policy. The researchers suggest that institutional strategies should prioritize capacity building over persuasion. Priorities include developing centralized infrastructure, providing targeted training, ensuring equitable access to resources, and offering clear financial and procedural guidance. Through these efforts, institutions can not only facilitate compliance but also foster a culture of responsible data stewardship and amplify the scientific and societal impact of research.

## Data Availability

All data associated with this article can be found in the supplementary materials.

## SUPPLEMENTAL FILES

**Appendix A:** Thematic Steps

**Appendix B:** Survey Instrument and Dataset
