# Supplementary material for: Supporting researcher compliance: assessing institutional readiness for the 2023 NIH Data Management and Sharing Policy
Source: J Med Libr Assoc. 2026 Oct 14;114(4):371–9. doi: 10.5195/jmla.2026.2361 (PMC13670228; doi:10.5195/jmla.2026.2361)
Supplement: Supplementary file 1 [file jmla-114-4-371-s01.docx]

**Initial Coding**

Key codes identified across the responses include:

| Initial code | n | % | Response IDs |
| --- | --- | --- | --- |
| **Desire for centralized/institutional support** | 12 | 52.2% | R1, R2, R6, R7, R9, R10, R12, R14, R17, R19, R22, R23 |
| **Need for expert consultation** | 9 | 39.1% | R2, R3, R7, R9, R10, R12, R13, R19, R22 |
| **Lack of clarity on policy/value** | 9 | 39.1% | R3, R7, R11, R13, R14, R18, R21, R22, R23 |
| **Training and templates needed** | 6 | 26.1% | R4, R7, R9, R10, R19, R20 |
| **Repository identification issues** | 5 | 21.7% | R2, R6, R10, R12, R14 |
| **Budgeting concerns** | 5 | 21.7% | R2, R5, R6, R14, R15 |
| **Time burden on PIs** | 4 | 17.4% | R1, R5, R16, R21 |
| **Technical barriers to data deposit (e.g., dbGaP, GEO)** | 4 | 17.4% | R10, R12, R18, R19 |
| **Uncertainty due to lack of experience** | 4 | 17.4% | R7, R11, R13, R23 |
| **Desire for in-house data infrastructure** | 3 | 13.0% | R6, R12, R17 |
| **Unclear responsibilities** | 3 | 13.0% | R11, R18, R22 |
| **Challenge of sharing PHI and clinical data** | 2 | 8.7% | R3, R12 |
| **Appreciation for collaboration** | 1 | 4.3% | R8 |

**Candidate Themes**

| Candidate theme | Description | n | % | Response IDs | % of all survey responses (55) |
| --- | --- | --- | --- | --- | --- |
| **1. Institutional Infrastructure and Support** | Researchers want DMS services integrated into institutional infrastructure. This includes staff support, templates, repositories, and consultations. | **15** | **65.2%** | R1, R2, R3, R4, R6, R7, R8, R9, R10, R12, R14, R17, R19, R22, R23 | 27.3% |
| **2. Burden and Capacity Concerns** | Researchers feel the DMS policy places a high burden on PIs, especially without dedicated support or expertise. | **6** | **26.1%** | R1, R5, R12, R16, R21, R22 | 10.9% |
| **3. Need for Clarity and Communication** | There's a strong desire for clearer institutional guidance and better communication about expectations, tools, and value of DMS. | **9** | **39.1%** | R3, R7, R11, R13, R14, R18, R21, R22, R23 | 16.4% |
| **4. Expertise and Equity in Access** | Inequities in access to support are a concern, as is availability of DMS-specific expertise. | **3** | **13.0%** | R11, R18, R19 | 5.5% |
| **5. Financial and Budgetary Constraints** | Concerns about costs, inflexible budgets, and unclear budgeting guidance related to DMS implementation. | **5** | **21.7%** | R2, R5, R6, R14, R15 | 9.1% |
| **6. Data Sharing Complexity** | Researchers see the technical aspects of preparing and depositing data (especially human data or PHI) as complex and lacking support. | **9** | **39.1%** | R3, R5, R6, R10, R12, R14, R18, R19, R22 | 16.4% |
| **7. Desire for Tailored and Practical Resources** | Participants ask for field-specific templates, curated examples, and applied help with tools like DMP Tool. | **6** | **26.1%** | R4, R7, R9, R10, R19, R20 | 10.9% |
| **8. Cultural and Attitudinal Resistance** | Some skepticism about institutional motives, DMS value, and overall policy burden reflects resistance to the change. | **2** | **8.7%** | R5, R21 | 3.6% |

**Mapping of Initial Codes to Candidate Themes**

Because responses could contain more than one idea, initial codes were not treated as mutually exclusive. As shown in the table below, some codes contributed to more than one candidate theme when they reflected both a practical implementation issue and a broader support or communication need.

| **Initial code** | **Candidate theme(s) informed** | **Rationale for mapping** |
| --- | --- | --- |
| Desire for centralized/  institutional support | 1. Institutional Infrastructure and Support | Reflected participants’ desire for institution-level systems, services, workflows, and centralized support for DMS compliance. |
| Need for expert consultation | 1. Institutional Infrastructure and Support; 4. Expertise and Equity in Access | Reflected the need for access to personnel with DMS-specific expertise and concerns that such expertise may not be equally available across investigators or departments. |
| Lack of clarity on policy/value | 3. Need for Clarity and Communication; 8. Cultural and Attitudinal Resistance | Reflected uncertainty about DMS expectations, communication gaps, and, in some cases, skepticism about the value or purpose of data sharing requirements. |
| Training and templates needed | 7. Desire for Tailored and Practical Resources; 1. Institutional Infrastructure and Support | Reflected requests for applied tools such as templates, examples, and training materials that could be provided through institutional support structures. |
| Repository identification issues | 6. Data Sharing Complexity; 1. Institutional Infrastructure and Support | Reflected practical difficulty identifying appropriate repositories and the need for institutional guidance or infrastructure to support repository selection. |
| Budgeting concerns | 5. Financial and Budgetary Constraints | Reflected concerns about DMS-related costs, grant budgeting, and uncertainty about allowable or sufficient funds for data sharing activities. |
| Time burden on PIs | 2. Burden and Capacity Concerns | Reflected concerns that DMS compliance adds workload and administrative responsibilities for investigators, particularly without dedicated support. |
| Technical barriers to data deposit | 6. Data Sharing Complexity; 4. Expertise and Equity in Access | Reflected challenges with technical platforms and data deposit processes, as well as the need for specialized expertise to navigate these systems. |
| Uncertainty due to lack of experience | 3. Need for Clarity and Communication; 4. Expertise and Equity in Access | Reflected uncertainty among researchers with limited prior DMS experience and the need for clearer guidance and accessible expertise. |
| Desire for in-house data infrastructure | 1. Institutional Infrastructure and Support; 6. Data Sharing Complexity | Reflected the need for local systems or institutional data infrastructure to reduce the practical complexity of preparing, storing, and sharing data. |
| Unclear responsibilities | 3. Need for Clarity and Communication; 2. Burden and Capacity Concerns | Reflected uncertainty about who is responsible for DMS tasks and how those responsibilities affect investigators’ workload. |
| Challenge of sharing PHI and clinical data | 6. Data Sharing Complexity | Reflected concerns about privacy, sensitive clinical data, de-identification, and the added complexity of sharing human subjects data. |
| Appreciation for collaboration | 1. Institutional Infrastructure and Support | Reflected positive recognition of institutional or collaborative support as helpful for navigating DMS requirements. |

**Final Overarching Themes**

**Theme 1: Operational and Infrastructure Needs**

1.1. Institutional Infrastructure and Support

1.2. Desire for Tailored and Practical Resources

1.3. Expertise and Equity in Access

1.4. Financial and Budgetary Constraints

**Theme 2: Implementation Challenges and Perceived Burden**

2.1. Burden and Capacity Concerns

2.2. Data Sharing Complexity

2.3. Need for Clarity and Communication

2.4. Cultural and Attitudinal Resistance
